# Development, application and evaluation of a 1-D full life cycle anchovy and sardine model for the North Aegean Sea (Eastern Mediterranean)

**DOI:** 10.1371/journal.pone.0219671

**Published:** 2019-08-15

**Authors:** Athanasios Gkanasos, Stylianos Somarakis, Kostas Tsiaras, Dimitrios Kleftogiannis, Marianna Giannoulaki, Eudoxia Schismenou, Sarantis Sofianos, George Triantafyllou

**Affiliations:** 1 Department of Environmental Physics, University of Athens, Athens, Greece; 2 Hellenic Centre for Marine Research (HCMR), Mavro Lithari, Anavyssos, Greece; 3 Hellenic Centre for Marine Research (HCMR), Thalassocosmos Gournes, Heraklion, Crete, Greece; 4 Genome Institute of Singapore (GIS), Agency for Science Technology and Research, Singapore; Universidad del Valle, COLOMBIA

## Abstract

A 1-D full-life-cycle, Individual-based model (IBM), two-way coupled with a hydrodynamic/biogeochemical model, is demonstrated for anchovy and sardine in the N. Aegean Sea (Eastern Mediterranean). The model is stage-specific and includes a ‘Wisconsin’ type bioenergetics, a diel vertical migration and a population dynamics module, with the incorporation of known differences in biological attributes between the anchovy and sardine stocks. A new energy allocation/egg production algorithm was developed, allowing for breeding pattern to move along the capital-income breeding continuum. Fish growth was calibrated against available size-at-age data by tuning food consumption (the half saturation coefficients) using a genetic algorithm. After a ten-years spin up, the model reproduced well the magnitude of population biomasses and spawning periods of the two species in the N. Aegean Sea. Surprisingly, model simulations revealed that anchovy depends primarily on stored energy for egg production (mostly capital breeder) whereas sardine depends heavily on direct food intake (income breeder). This is related to the peculiar phenology of plankton production in the area, with mesozooplankton concentration exhibiting a sharp decrease from early summer to autumn and a subsequent increase from winter to early summer. Monthly changes in somatic condition of fish collected on board the commercial purse seine fleet followed closely the simulated mesozooplankton concentration. Finally, model simulations showed that, when both the anchovy and sardine stocks are overexploited, the mesozooplankton concentration increases, which may open up ecological space for competing species. The importance of protecting the recruit spawners was highlighted with model simulations testing the effect of changing the timing of the existing 2.5-months closed period. Optimum timing for fishery closure is different for anchovy and sardine because of their opposite spawning and recruitment periods.

## Introduction

Small pelagic fishes (SPF), like anchovies and sardines, are short-lived, highly fecund, planktivorous fishes that play a key role in marine food webs and are very important for fisheries and human communities worldwide [1]. They are very sensitive to environmental changes and extremely variable in their abundance at both inter-annual and inter-decadal scales ([2], [3]). An effective management system for these resources would require better understanding of the mechanisms controlling rapid variations in abundance and productivity of populations, and the consequences that these variations may have for ecological interactions ([4], [5]).

In European waters, stocks of SPF have historically exhibited large variations in abundance but, in contrast to the Northwest Pacific and in Eastern boundary currents, co-occurring European anchovy *Engraulis encrasicolus* and European sardine *Sardina pilchardus* stocks have not exhibited large, out-of-phase fluctuations [6]. In the Mediterranean Sea, most anchovy and sardine stocks have been declining in recent years (e.g. [7], [8]; [9], showing also decreasing trends in maximum size and somatic condition ([10], [11]). For example, in the Gulf of Lions, where fishing pressure on anchovy and sardine stocks is very low, the reductions in biomass, body condition and maximum size/age have been attributed to increasing temperature and reduced water mixing, affecting planktonic productivity ([10], [11], [12]).

**Fig 1:**
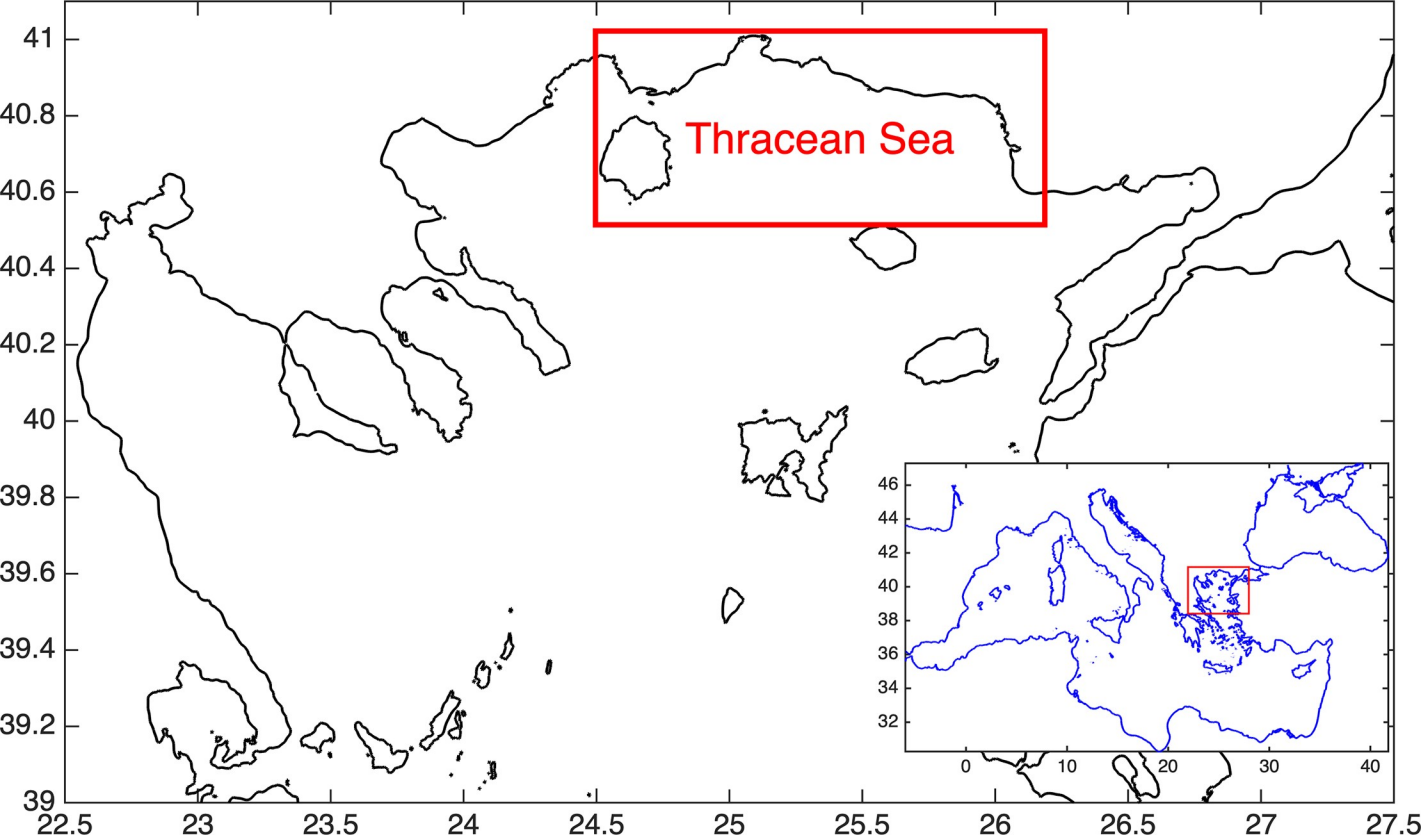
Map of the North Aegean Sea showing the model domain. The box indicates the location of Thracian Sea.

The aim of the present study was to develop a multispecies (anchovy-sardine) full life cycle, individual based model (IBM) for stocks inhabiting the N. Aegean Sea (Eastern Mediterranean) (Fig 1). Full-life-cycle, bioenergetics IBMs, coupled with hydrodynamic/biogeochemical models allow for a mechanistic understanding of how the physics, biogeochemistry, and biology combine to result in patterns of variability in growth, egg production, recruitment and spawning stock biomass ([6], [13], [14]).

For European anchovy, coupled bioenergetics or bioenergetics-IBMs have been successfully implemented in the Black Sea [15], the Bay of Biscay ([16], [17]), the North Aegean Sea ([18], [19]) and the Gulf of Lions [20]. A European sardine model has also been developed in the Bay of Biscay [17]. These models were based on either the ‘Wisconsin’ [21] or the Dynamic Energy Budget (DEB) [22] framework, and they were offline or, occasionally, online coupled with regional hydrodynamic-biogeochemical models. They were generally implemented in a 1-D configuration, thus lacking a horizontal movement module, except for a 3-D application to the N. Aegean Sea anchovy stock [19].

1-D models lack the horizontal dimension, i.e. a movement/migration module, yet they comprise an initial step useful for calibrating growth, egg production and/or population biomass to the average thermal and trophic conditions of the ecosystem (e.g. [23], [24]). They have also been used effectively in basin-scale or latitudinal comparisons between stocks (e.g. [25], [26]). Finally, 1-D IBMs provide a means to test straightforwardly the outcomes of management measures (e.g. temporal fishing bans, reductions of fishing mortality), especially in the Mediterranean Sea where the collection of spatially explicit fisheries data has only recently been started and the utility of the collected information has often been questioned [27].

The main biological differences between anchovy and sardine in the N. Aegean Sea include their reproductive traits (winter spawning, low daily fecundity in sardine–summer spawning, high daily fecundity in anchovy ([28], [14]) and the generally longer life span and maximum size of sardine [29]. On the other hand, the two stocks have many similarities, i.e., high diet overlap, closely correlated diel feeding patterns/food consumption rates ([30], [31], [32]), and similar diel vertical migration behavior ([33], [34]). Finally, temperature optima for growth are almost identical for the two species, at least during the juvenile stage ([35], [36], [14]).

The Mediterranean sardine is considered to be primarily a capital breeder, i.e. it stores energy and uses it later for egg production ([37], [38], [14]). In contrast, the Mediterranean anchovy is thought to be more close to the income breeding pattern, i.e. egg production is mainly fueled by direct food intake during the spawning period ([39], [14]). Breeding pattern has consequences for recruitment [14] and coupled bioenergetics models provide capability for directly assessing it, by linking energy acquisition and allocation to egg production to the seasonal cycle of food production (zooplankton) as simulated by the biogeochemical model [17].

The IBM model for anchovy and sardine presented in this paper was based on an existing model for anchovy in the N. Aegean Sea [19]. We have built a new energy allocation/egg production algorithm that allows for breeding pattern to move along the capital-income continuum (*sensu* [17]). Fish growth was calibrated against available size-at-age field data using a genetic algorithm. Finally, the model was used to test the outcomes of different management measures, such as changes in the exploitation rate of the stocks as well as shifts in the timing of an existing fishery ban (closed period for the purse seine fishery: 15 Dec–Feb, [40]).

## Materials and methods

### Low trophic level model

The fish model is on-line, two-way coupled with a 1-D (water column) lower trophic level model (LTL) implemented in the Thracian Sea (Fig 1). The Thracian Sea is one of the major habitats of anchovy and sardine in the Aegean Sea ([41], [42], [43]).

**Fig 2:**
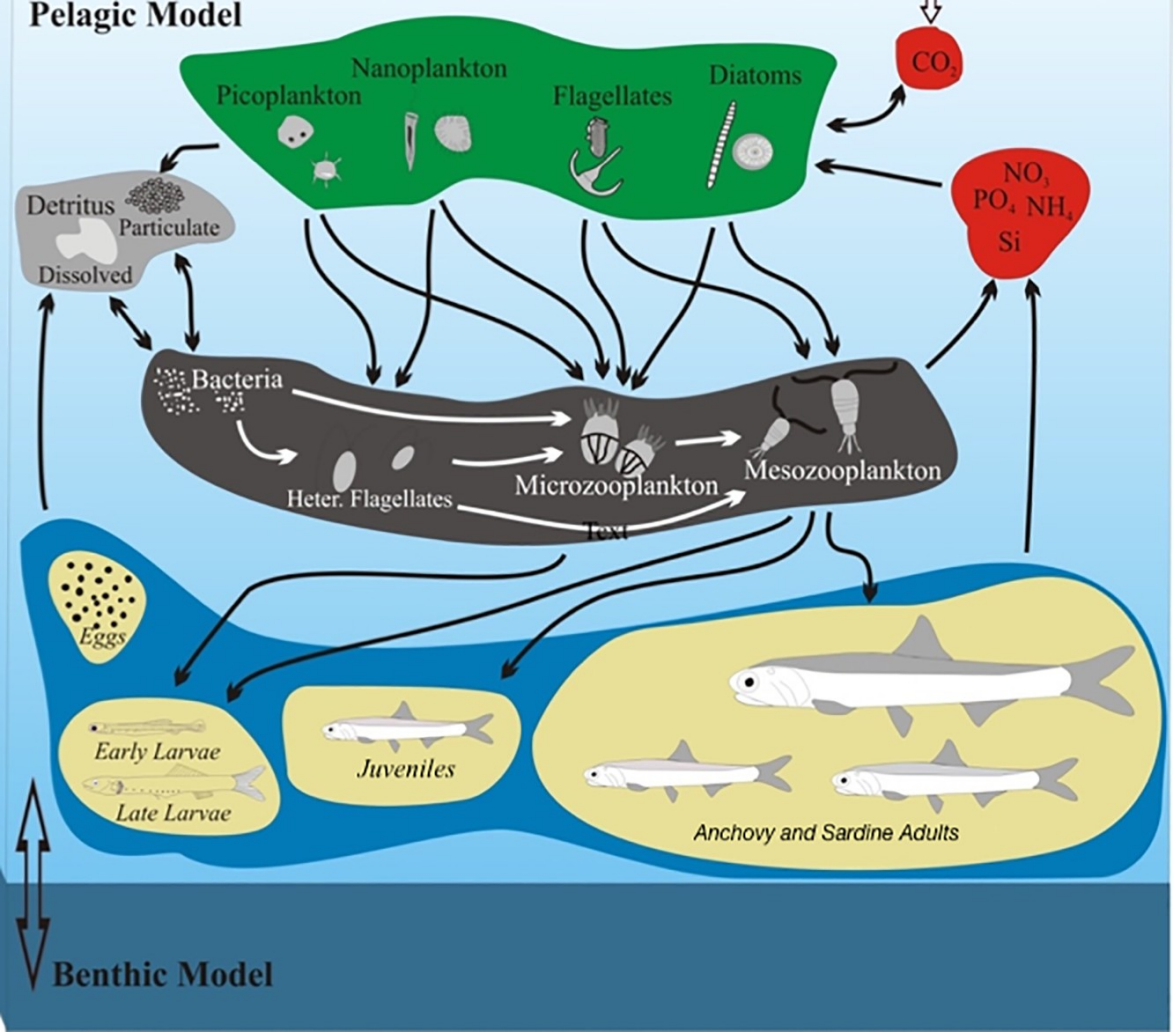
Representation of the anchovy and sardine model coupled with the lower trophic level (LTL) model.

The LTL provides the prey fields (zooplankton) and temperature conditions to the fish model (Fig 2) and consists of a hydrodynamic model, based on POM (Princeton Ocean Model; [44]) and a biogeochemical model, based on the European Regional Seas Ecosystem Model (ERSEM, [45]). It has already been implemented in the Cretan Sea [46], the North Aegean Sea ([47], [48]), as well as the entire Mediterranean Sea, as part of the POSEIDON forecasting system (www.poseidon.hcmr.gr). The ERSEM follows the functional group approach, with organisms being classified according to their trophic role (producers, consumers, etc.) and size. It describes the planktonic food web with four groups of primary producers (picophytoplankton, nanophytoplankton, diatoms, dinoflagellates), bacteria, and three zooplankton groups (heterotrophic nanoflagellates, microzooplankton, mesozooplankton), as well as the particulate and dissolved organic matter pools. Carbon dynamics are coupled with nitrogen (nitrate, ammonium), phosphorus (phosphate) and silicate cycles, with all plankton groups having dynamically varying C:N:P:Si pools.

The biogeochemical model is forced by temperature and daily vertical diffusivity profiles, averaged for the 2003–2008 period, over the Thracian Sea. These were obtained off-line from a 3-D simulation of the hydrodynamic model [48]. Given that the coupling with hydrodynamics is only one-way, using the full 3-D hydrodynamic output was preferable. A 1-D hydrodynamic model does not resolve horizontal processes and has important limitations in this area where lateral water inputs (Black Sea Water, rivers etc) are very important. Water column properties (temperature, salinity) are therefore not realistically simulated with a 1-D hydrodynamic model. A monthly varying input of dissolved inorganic nutrients (phosphate, nitrate, ammonium, silicate) was adopted at the surface layer to mimic river/Black Sea Water (BSW) nutrient inputs in the Thracian Sea. This nutrient input follows the seasonal variability of riverine/BSW inputs, peaking during spring and is tuned so that the simulated plankton productivity (Chl-a, zooplankton) is similar to the one simulated with the 3-D version of the biogeochemical model [48].

### Fish model

The fish model is a full-life cycle, individual based model (IBM) and includes two species, the European anchovy (*Engraulis encrasicolus*) and the European sardine (*Sardina pilchardus*). It was based on the anchovy model developed by Politikos [19]. The sardine IBM was built from the existing anchovy model by progressively integrating traits that are known to differ between the two species (Table 1).

**Table 1:** Main differences and similarities in model parameters between anchovy and sardine.

Parameter		Anchovy	Sardine
Length range (mm), [49]	Early larvae	4–11	5–13
	Late larvae	11–42	13–50
	Juvenile	42–100	50–105
Length at maturity (L_m,_ mm), [49]		100	105
Egg energy, [17]		0.66	1.11
Daily specific fecundity (eggs g^-1^), [42], [28]		46	20.1
Batch Energy (g prey per g fish per day)		0.012	0.0086
Spawning period SST threshold, [50], [51]		SST >15°C	SST <16°C
Natural mortalities, [19], [52], [53] The natural mortality of juveniles was calibrated (see text for details).		0.4, embryos	
		0.2, early larvae	
		0.05, late larvae	
		0.012, juveniles	
		0.002, adults	
Fishing mortalities, [19], [52], [53]		0.00136, adults	0.002, adults
Reference biomass (t), [19], [52], [53]		40000	25000

The model describes the life cycle of both species, from the egg to the adult stage. The life span is divided into seven stages/age classes for anchovy (embryo, early larva, late larva, juvenile, adult age-1 to age-3) and eight stages for sardine (with an additional adult age class: adult age-1 to age-4) (Table 1). The number of age classes was defined based on otolith age readings made on samples collected in the field ([42], see below).

Although this version of the multispecies model is 1-D, i.e. it lacks a horizontal movement algorithm, it includes all other modules described in [19], namely a bioenergetics, a diel vertical migration (DVM) and a population module. The populations of the two species are represented by a fixed number of super-individuals (SIs) [54], in each stage/age-class. Each SI consists of individuals that share the same attributes (length, weight, age etc.). During a spawning event, a new (egg) SI is created. For computational efficiency, the maximum number of SIs per stage is maintained constant throughout the simulations. It is higher (150 SIs) for the early life stages (embryos, early and late larvae) and lower for the juvenile stage and adult age classes (10 SIs). A higher number of SIs was necessary for the egg and larval stages in order to resolve adequately the dynamics of these stages during the prolonged spawning periods of the two species.

Fish growth is calculated with a Wisconsin-type bioenergetics model taking into account all important physiological processes, i.e. consumption, respiration, egestion, specific dynamic action, excretion and reproduction (Table 2). A piece-wise length-weight relationship is used to convert weight to length (see [18] for details).

**Table 2:** Equations and parameters of the bioenergetics model.

Energy Process	Equations	Parameters Anchovy	Parameters Sardine
Somatic growth	$\frac{1}{W_{SI}}∙\frac{dW_{SI}}{dt}=[C-(R+EG+SDA+EX+E_{buffer})]∙\frac{{CAL}_{z}}{{CAL}_{f}}$		
	W_SI_ = fish wet weight (g), t = time (days), C = consumption, R = respiration, EG = egestion, SDA = specific dynamic action, EX = excretion, E_buffer_ = the energy allocated to reproduction, CAL_z_ = caloric equivalent of zooplankton, CAL_f_ = caloric equivalent of fish		
Maximum Consumption (C_max_)	$C_{max}=a_{c}W_{SI}^{b_{C}}f_{C}(T)$, *f*_*C*_(*T*) = *V*^*X*^*e*^*X*(1−*V*)^, a_c_ = Intercept for consumption, b_c_ = Exponent for consumption	a_c_ = 0.41, b_c_ = 0.31	
Temperature function	$V=\frac{T_{max}-T}{T_{max}-T_{opt}},$ *S* = (*lnQ*_*c*_)(*T*_*max*_−*T*_*opt*_), *Y* = (*lnQ*_*c*_)(*T*_*max*_−*T*_*opt*_+2), $X=\frac{S^{2}{(1+{\left(1+40/Y\right)}^{1/2}}^{2}}{400}$, Q_c_ = Slope for temperature dependence, T_opt_ = Optimum Temperature (^o^C), T_max_ = Maximum Temperature (^o^C)	Q_c_ = 2.22^a^^,^^b^, 2.4^c^^,^^d^, T_opt_ = 17.25^a^,16.25^b^,15.8^c^^,^^d^, T_max_ = 27	T_opt_ = 14.5^a^,14.75^b^,15.8^c^^,^^d^
Consumption (C)	$Ci=\sum_{i=1}^{2}C_{j,i},\;C_{j,i}=\frac{C_{max}(\frac{{PD}_{j,i}v_{j,i}}{k_{j,i}})}{1+\sum_{i=1}^{2}(\frac{{PD}_{k,i}v_{k,i}}{k_{k,i}})}$, PD_j,i_ = density of prey type i (i = 1 corresponds to microzooplankton and i = 2 to mesozooplankton) (g-prey m^-3^) for life stage/age class j, v_j,i_ = vulnerability of prey type i to life stage/age class j (dimensionless), k_j,i_ half saturation function (g-prey m^-3^) for life stage j feeding on prey type i.	v_2,1_ = 1.0, v_3,1_ = 0.5, v_4,1_ = v_5,1_ = v_6,1_ = v_7,1_ = 0, v_2,2_ = 0.0, v_3,2_ = 0.5, v_4,2_ = v_5,2_ = v_6,2_ = v_7,2_ = 1.0	
Respiration (R)	$R=a_{r}W_{SI}^{b_{r}}f_{R}(T)A,\;f_{R}(T)=Q_{10}^{\frac{T-T_{m}}{10}},\;A=e^{d_{r}U},\;U=a_{A}W^{b_{A}}e^{\left(C_{A}T\right)}$, a_r_ = Intercept for respiration, b_r_ = Exponent for respiration, Q_10_ = Temperature dependence parameter, T_m_ = Mean annual temperature, d_r_ = Coefficient for R for swimming speed, a_A_ = Intercept U (< 12.0 ^o^C), a_A_ = Intercept U (≥12.0 ^o^C), a_A_ = Intercept U (≥12.0 ^o^C), (during low feeding activity), b_A_ = Coefficient U for weight, c_A_ = Coefficient U vs. temperature (< 12.0 ^o^C), c_A_ = Coefficient U vs. temperature (≥12:0 ^o^C)	a_r_ = 0.003, b_r_ = 0.34, Q_10_ = 1.3, T_m_ = 16^a^^,^^b^^,^^c^^,^^d^, d_r_ = 0.022, a_A_ = 2.0 (U< 12.0 ^o^C), a_A_ = 12.25^a^^,^^b^, 11.98^c^, 14.21^d^ (U≥12.0 ^o^C), a_A_ = 9.97^c^ (U≥12.0 ^o^C), (during low feeding activity), b_A_ = 0.27 ^a^^,^^b^, 0.33^c^, 0.27^d^, c_A_ = 0.149 (U< 12.0 ^o^C), c_A_ = 0.0 (U≥12:0 ^o^C)	
Egestion (EG)	*F* = *a*_*f*_*C*, a_f_ = Proportion of food egested	a_f_ = 0.15^a^^,^^b^, 0.126^c^^,^^d^	
Excretion (EX)	*E* = *a*_*e*_(*C*−*F*)+*b*_*e*_, a_e_ = Excretion coefficient, b_e_ = Proportion of food excreted	a_e_ = 0.41, b_e_ = 0.01	
Specific Dynamic Action (SDA)	*SDA* = *a*_*sda*_(*C*−*F*), a_sda_ = Specific dynamic action coefficient	a_sda_ = 0.10	
Length-weight relationship	y = b_o_+b_1_x+b_2_(x-d_1_)(x>d_1_)+b_3_(x-d_2_)(x>d_2_), y, x (log-transformed fish wet weight and length), b_o_ = y-intercept, b_1_ = slope of the function for the larval stage, b_2_ = slope change for the juvenile stage, d_1_ = slope change inflexion point, b_3_ = subsequent slope change for the adult stage, d_2_ = corresponding length for this slope respectively	b_o_ = -6.1158, b_1_ = 3.5764, b_2_ = -0.616, d_1_ = 1.5798, b_3_ = 0.7137, d_2_ = 1.954	b_o_ = -9.229, b_1_ = 5.391, b_2_ = -2.281, d_1_ = 1.699, b_3_ = 0.106, d_2_ = 2.02

^a^ Early larval stage (j = 2).

^b^ Late larval stage (j = 3).

^c^ Juvenile stage (j = 4).

^d^ Adult age-classes (j = 5,6,7 & 8 for sardine).

As already mentioned, the fish model is on-line coupled with the LTL model (Fig 2). Early larvae feed on microzooplankton, late larvae consume micro- and mesozooplankton and juveniles/adults interact only with the mesozooplankton compartment of the ERSEM. The plankton biomass (micro- and mesozooplankton) that is consumed by the fish is removed in ERSEM, while fish bio-products from egestion, excretion and specific dynamic action are directed to the ERSEM particulate organic matter and dissolved inorganic nutrient pools. The individuals that each SI represents are assumed to have a vertical distribution (position in the water column) that is maximized around the depth of peak prey availability. Eggs and early larvae are distributed in the surface layer (0-30m), while late larvae, juveniles and adults perform diel vertical migrations between the surface (0-30m, night) and the sub-surface (>30m, day). In order to predict the duration of the embryonic stages (egg+yolk sac larva), which are temperature dependent, we use the equations developed by [17].

The number of individuals in each SI is computed by taking into account the natural and fishing mortality. Specifically, at each time step, the number of individuals within each SI (*N*) is reduced using the equation:

$$\frac{dN}{dt}=-(M+F)\times N$$

where *M* is the assigned natural mortality and *F* is the fishing mortality rate, applied only to adult SIs (see Table 1).

For the embryonic and larval stages, the adopted M values for anchovy were based on published estimates ([41]; [55]; [42]). In the case of European sardine, literature information was very limited. The few existing values for egg and early larval mortality, estimated for the Iberian sardine in the Atlantic ([56], [57]) were very similar to the values adopted for the Mediterranean anchovy. We therefore used the same values of natural mortality for the early life stages of the two species (Table 1).

The natural mortality during the juvenile stage is largely unknown. Yet, mortality during the juvenile stage has a great impact on subsequent population biomass due to the stage’s long duration. The natural mortality rate of juveniles was therefore calibrated, so as the simulated anchovy and sardine populations to fluctuate around 40000 t and 25000 t respectively, which are approximate mean biomasses of the two species in the N. Aegean Sea (based on acoustic data biomass estimations for the period 2003–2008 ([52], [53], Table 1). The mean natural and fishing mortalities of adults were adopted from the aforementioned stock assessment papers (Table 1). Except from natural and fishing mortalities, additional starvation mortality is imposed for all stages (i.e., the SI vanishes) in case that the cumulative weight loss exceeds 35%. This 35% threshold was defined empirically based on residual variation of existing length-weight relationships (see [19] for details).

**Fig 3:**
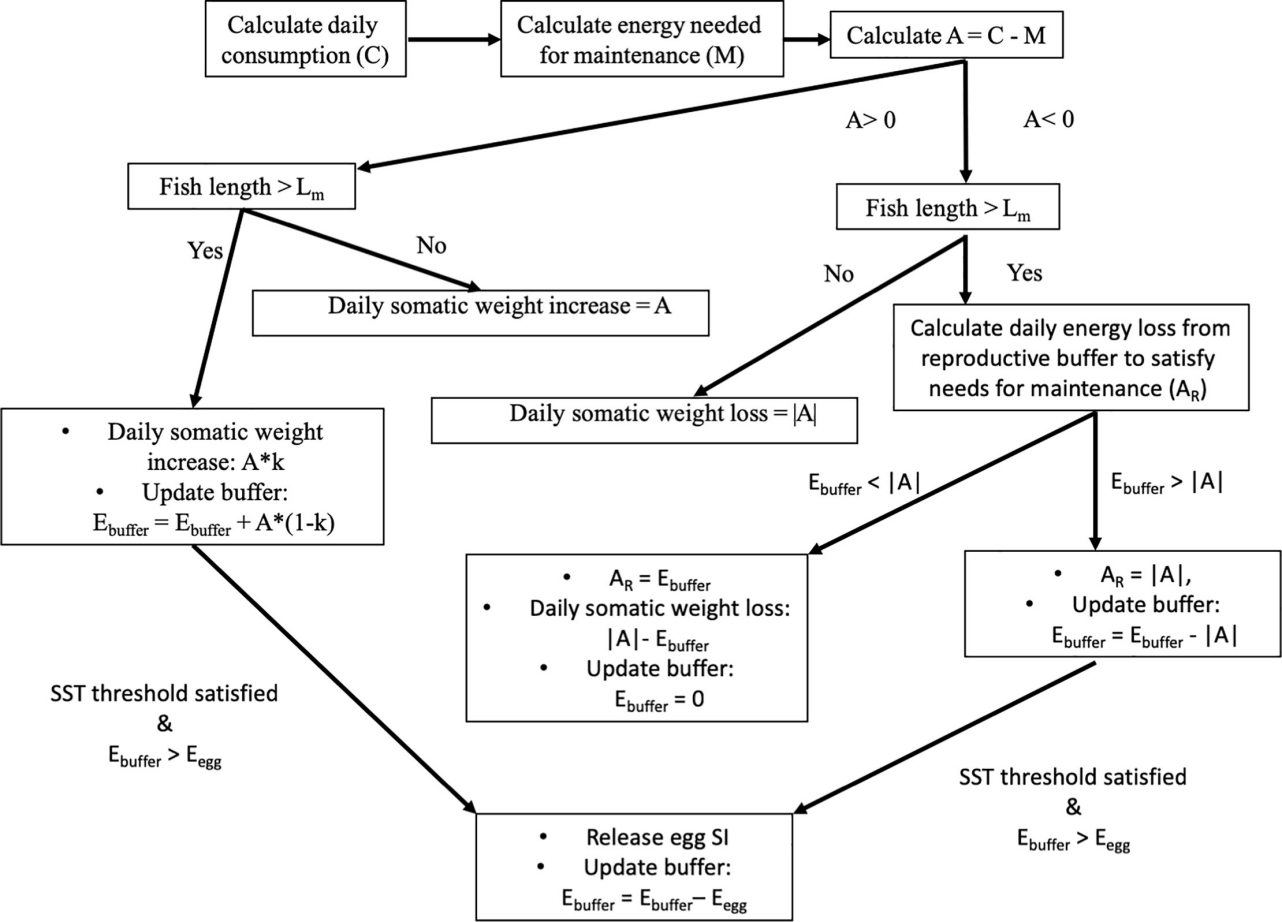
Schematic illustration of the energy allocation and egg production algorithm. SST: Sea surface temperature. L_m_: length at maturity. E_buffer_: energy in reproduction buffer. E_egg_: batch energy.

Spawning is regulated by an energy allocation/egg production algorithm, embedded in the bioenergetics equation (Fig 3). This algorithm is different from the one described in [19]. The latter assumed an extreme income breeding mode for the Mediterranean anchovy. The new algorithm (Fig 3) is now allowing for breeding pattern to move along the capital-income continuum [38]. A similar approach was followed in [17]. Briefly, the energy available from consumption is first used to satisfy the needs of maintenance (M) that accounts for respiration, egestion, specific dynamic action, excretion. The remainder energy (A) is then channeled to only growth (increase in weight), if fish is smaller than length at maturity (L_m_). This is justified from measurements in European sardine showing that, in juvenile fish, growth is prioritized and immature fish do not store fat [58]. If fish is larger than L_m_, the surplus energy (A) is channeled to both growth and reproduction. Energy allocated to reproduction is stored, all year round, in the so-called ‘reproductive buffer’ [16]. The amount of A allocated to reproduction is (1-k)*A. The parameter k is largely unknown and therefore assumed to be k = 0.5 in both species. If A<0, energy already in the reproductive buffer (first) and fish soma (secondly) goes to maintenance (to meet daily maintenance costs) (Fig 3). Regarding spawning, each SI releases an egg batch (egg SI) on a daily basis, if a (species specific) SST criterion is satisfied, fish length is larger than L_m_ and energy stored in the buffer (E_buffer_) is sufficient for producing the egg batch.

The number of eggs released (the population of the egg SI) is equal to the product of daily specific fecundity (DSF, number of eggs per gram of the adult SI) and the SI’s weight. Different values of DSF were adopted for anchovy and sardine, based on published literature ([42], [28]). The batch energy (E_egg_) is calculated from DSF and egg energy. We used the values of anchovy and sardine egg energy calculated in [17] (Table 1).

Based on the fact that the two species have different spawning periods in the Mediterranean Sea, with anchovy spawning from spring to autumn and sardine from autumn to spring [14], anchovy is set to spawn when sea surface temperature (SST) is above 15°C [50] while sardine spawns only if SST <16°C [51]. Given the different spawning periods, we also adopted different optimum temperatures for larval consumption ([6], Table 2). These were selected so as to lay close to the actual average temperatures that larvae experience. Apart from SST, an additional criterion (not shown in Fig 3) was also applied to define the end of the spawning period. It is known that, in the lack of food, fish stop releasing eggs and start to absorb their gonads (a process known as atresia). If food shortage is prolonged (8–9 days in Northern anchovy) the spawning period of the fish comes to an end [59]. We therefore assumed that if food consumption is insufficient to meet metabolic requirements for 9 consecutive days the SI stops releasing eggs for that particular spawning season.

Field data for the construction and calibration of the fish model included length-weight measurements and length/weight-at-age estimates. For anchovy, these data are described in [18], [19] and [26]. For sardine, we used data available from [49] and [36] for larvae and juveniles, and data from the acoustic and daily egg production surveys carried out in the N. Aegean Sea from 2003 to 2008 [42].

Additionally, we studied the monthly variation of the somatic condition of two species using length-weight measurements made on fish collected from the commercial purse seine fleet from 2003 to 2008 (S1 File). No samples were available for January and February, which is a closed period for the purse seine fishery [40]. Size-adjusted monthly mean weights were estimated for each species using a general linear model approach (S1 File). The rationale for studying the monthly variation of fish condition (which reflects energy storage [see [11] and references therein] was to compare its changes with model predictions for the seasonal zooplankton cycle and fish breeding patterns (income-capital).

For this purpose, a ‘capital index’ similar to the one developed in [17] was computed for each age class:

$$(dE_{buffer}-\sum_{s}^{e}A_{R})/\sum_{s}^{e}E_{egg}$$

It corresponds to the quotient of the division between the energetic loss from the reproductive buffer between the start (s) and the end (e) of the spawning season (after the subtraction of the cumulative emergency maintenance costs paid from the reproductive buffer, as described in Fig 3) and the cumulative energy spent for egg production during the spawning season. The higher is the capital index, the closer is the species to the capital breeding pattern, i.e. it is more dependent on stored energy for the production of eggs.

### Calibration of the bioenergetics model

The bioenergetics module was calibrated against the available length- and weight-at-age field data by applying a heuristic optimization technique based on a genetic algorithm (GA). GAs are inspired from the principles of natural selection and they are effective when dealing with large and complicated search spaces or when there is no other analytical solution for the problem. GAs are often characterized as population based evolutionary processes, starting with a population of candidate solutions (called chromosomes) that are evolved in time via a number of cycles (called generations) and genetic operations (i.e., crossover and mutation) towards a specific goal that is described by a problem-specific optimization function (called fitness function). Chromosomes consist of genes, which in our application are the model parameters to be tuned. For every generation, the fitness function is evaluated for every chromosome estimating in this way the quality of the candidate solution (e.g., highest score indicates better solution). While passing from one generation to another, solutions that achieve the highest score are selected to survive. The process is continued until some termination criteria are fulfilled or a user-defined number of generations is reached [60]. Here, for simplicity and in order to achieve reproducibility of our results, we deployed a simple genetic algorithm adopted by the implementation described in [61].

Since the objective was to tune the model and achieve average weights-at-age for each species as close as possible to field data, we introduced a simple fitness function:

$$Fitness=1/\sqrt{\sum {\left(prediction-reference\right)}^{2}}$$

This fitness function takes into account the Euclidean distance between weight data and the predicted weight of the species for one or more predefined dates. Thus, given two weight outputs for a specific age, derived from different model runs using different parameter values, the higher ranked output is the one that has smaller distance with respect to the reference weight.

Regarding the termination criteria, we set a maximum number of generations equal to 1000. To reduce the execution time, the algorithm is equipped with an additional stopping criterion, which considers the population as converged (i.e., steady-state) if there is no difference in the average fitness value of the population for 150 consecutive generations.

The genetic algorithm was applied to the stage-/age-specific half saturation coefficients (see equation for consumption in Table 2) that regulate food consumption [26]. For this purpose, we used an IBM version that included only one super-individual from each species. By experimenting with the GA setup, we compared a simultaneous parameter tuning (all stage-/age-specific half saturation coefficients) to a sequential parameters tuning. The later involved the tuning of the half saturation constant first for the larvae, then for the juveniles etc. until the terminal adult age class. The sequential tuning approach proved more successful in predicting the available growth data.

### Model simulations setup and testing of management measures

The anchovy-sardine IBM, with a time-step 1200 sec, was run for a 30 year period in order to evaluate its performance in terms of population and reproductive characteristics of the two species in the North Aegean Sea. The first ten years were considered as model spin up. Hence, only the remaining period (11–30 years) was taken into account for model evaluation and analysis.

Subsequently, we used the model to test the sensitivity of the anchovy and sardine populations to (a) changes in fishery exploitation rates, and (b) changes in the timing of the existing 2.5 months closure period.

In the first set of simulations, we examined the effect of changing the levels of fishing mortality on the populations of anchovy and sardine as well as on their mesozooplankton prey. The fishing mortality of each species was allowed to vary, so that the Paterson’s exploitation rate (E.R. = fishing mortality/[natural mortality + fishing mortality]) fluctuated around 0.4 (0.23 to 0.51 and 0.32 to 0.46 for anchovy and sardine respectively). The value of 0.4 (as empirically defined by [62]), is currently considered as the exploitation rate corresponding to the maximum sustainable yield for the Mediterranean small pelagic fish stocks [63] and is a reference point for their management, i.e. stocks exploited above 0.4 are considered overexploited.

In the second set of simulations, we examined the effect of changing the timing of the existing 2.5 months purse-seine fishery ban, now scheduled between 15 December and end of February, by shifting it by one month along the year, i.e. 15 January-March, 15 February-April etc.

## Results

**Fig 4:**
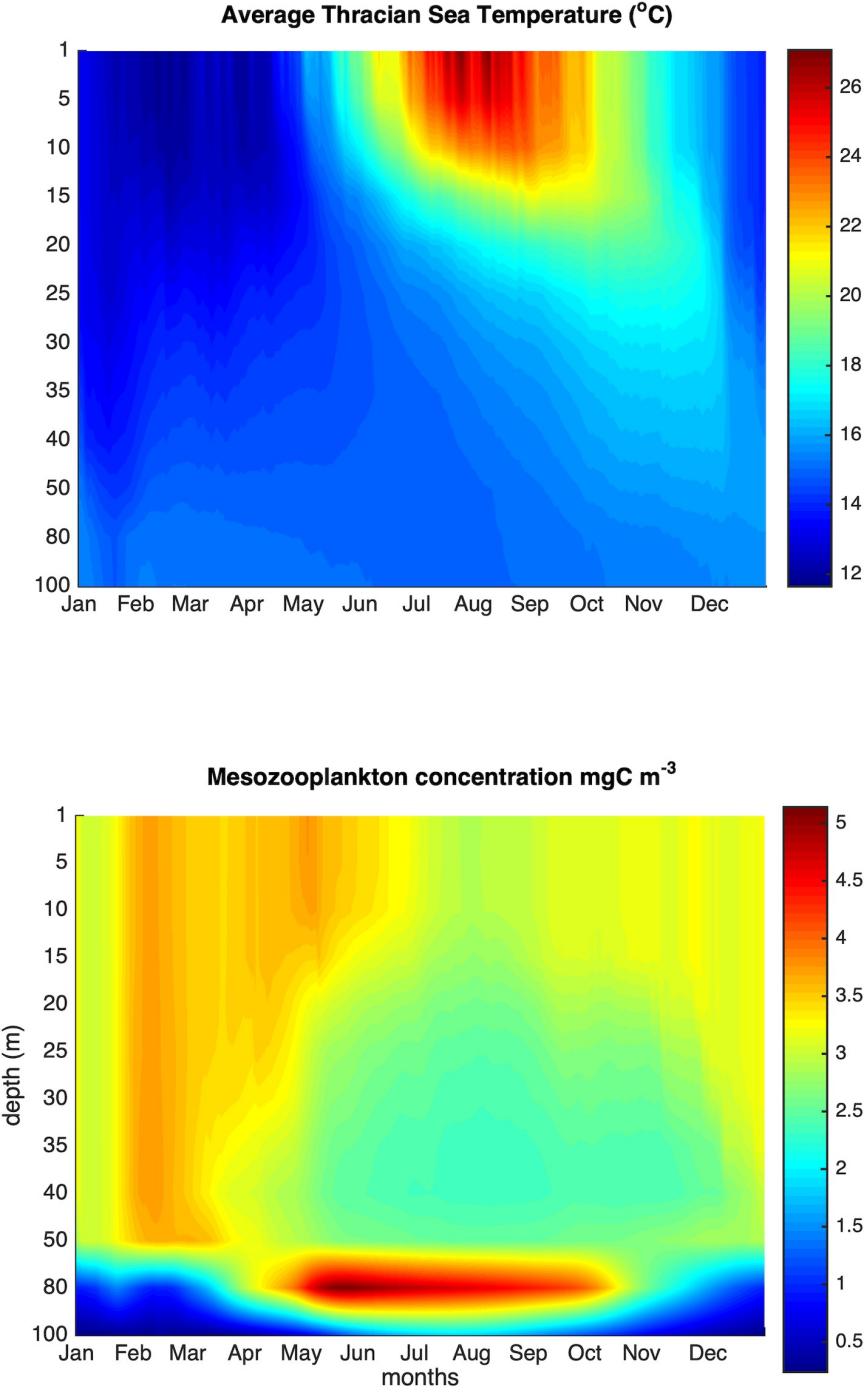
Seasonal evolution of temperature and simulated mesozooplankton concentration.

The seasonal variability of the water column temperature and mesozooplankton concentration is shown in Fig 4, highlighting the development of a strong thermal stratification during summer, coupled with the formation of a deep mesozooplankton maximum (corresponding to the deep chlorophyll maximum). The simulated mesozooplankton concentration is comparable with that of the 3-D model output [48] that has been validated against *in situ* data [64].

**Fig 5:**
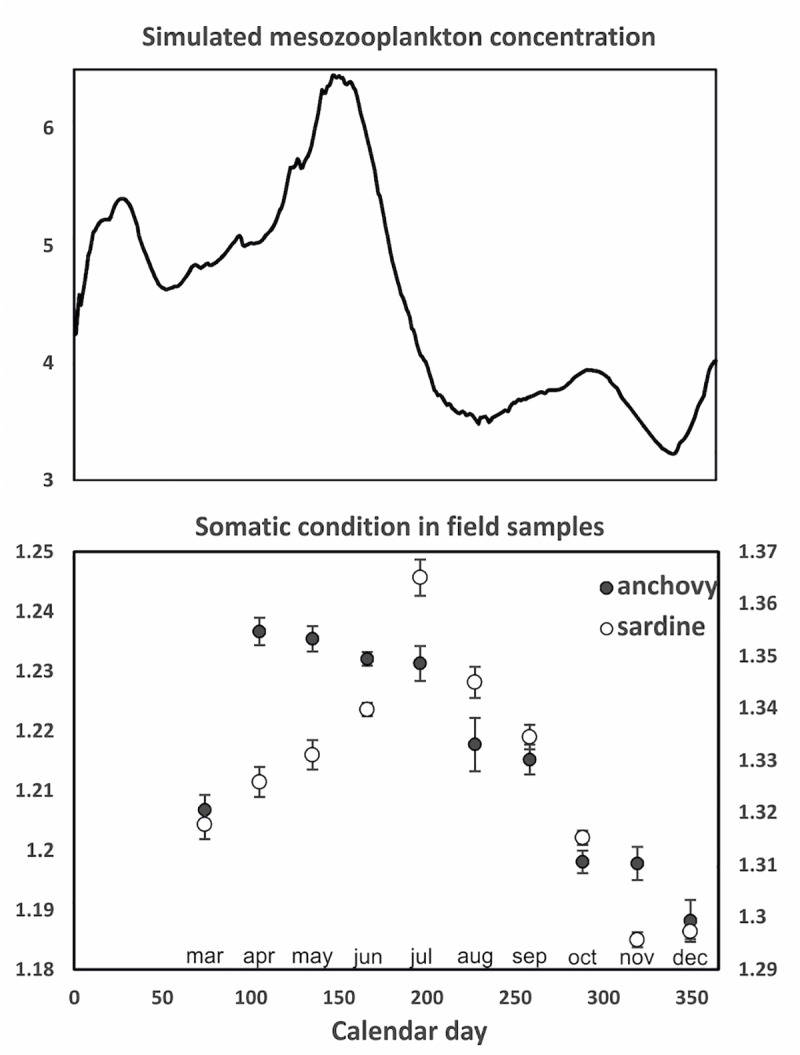
Top panel: Simulated average mesozooplankton concentration (mgC m^-3^) in the water column (0-100m) against calendar day. Bottom panel: length-adjusted monthly mean weight (somatic condition) of fish samples collected onboard the Thracian Sea purse seine fleet in 2003–2008.

Starting from the onset of the mixing period in winter, the mean mesozooplankton concentration exhibits an increasing trend that lasts till early summer (Fig 5). Thereafter, it decreases sharply and remains low until mid-December. The mean monthly somatic condition of anchovy and sardine in the Thracian Sea (estimated from the field samples, S1 File) appears to follow closely the seasonal variability of the simulated mesozooplankton concentration (Fig 5). Although no samples were available in the January-February period to estimate somatic condition, results showed that the latter increased from December to spring in both species (more sharply in anchovy with the summer spawning period, more slowly in the winter spawning sardine). Interestingly, somatic condition starts to decrease sharply after July, i.e. approximately one month after the strong decrease in the simulated mesozooplankton concentration.

**Fig 6:**
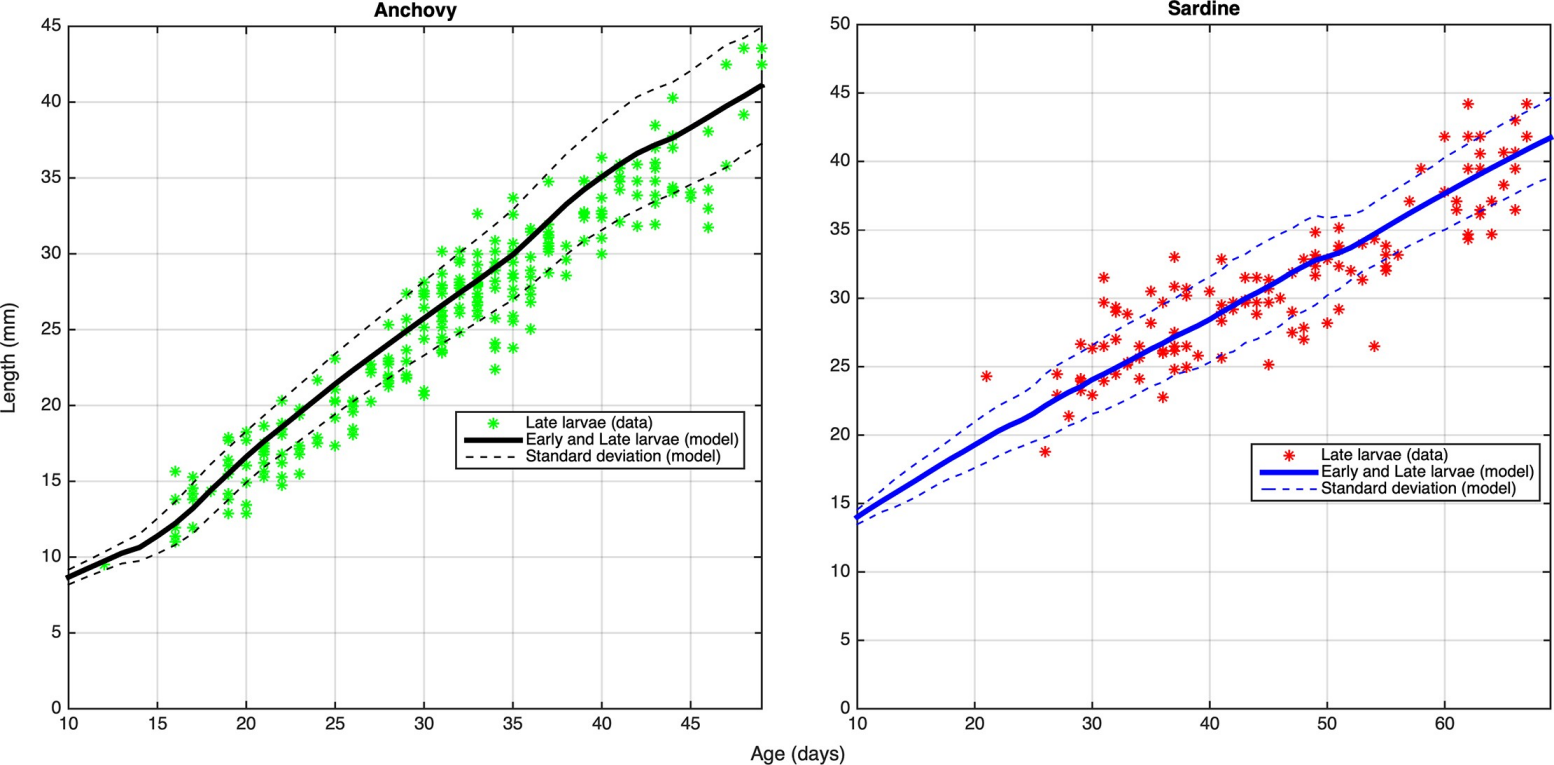
Mean length-at-age (±SD) of anchovy and sardine larvae, calibrated using the genetic algorithm and field data ([49], [65], [36]).

**Fig 7:**
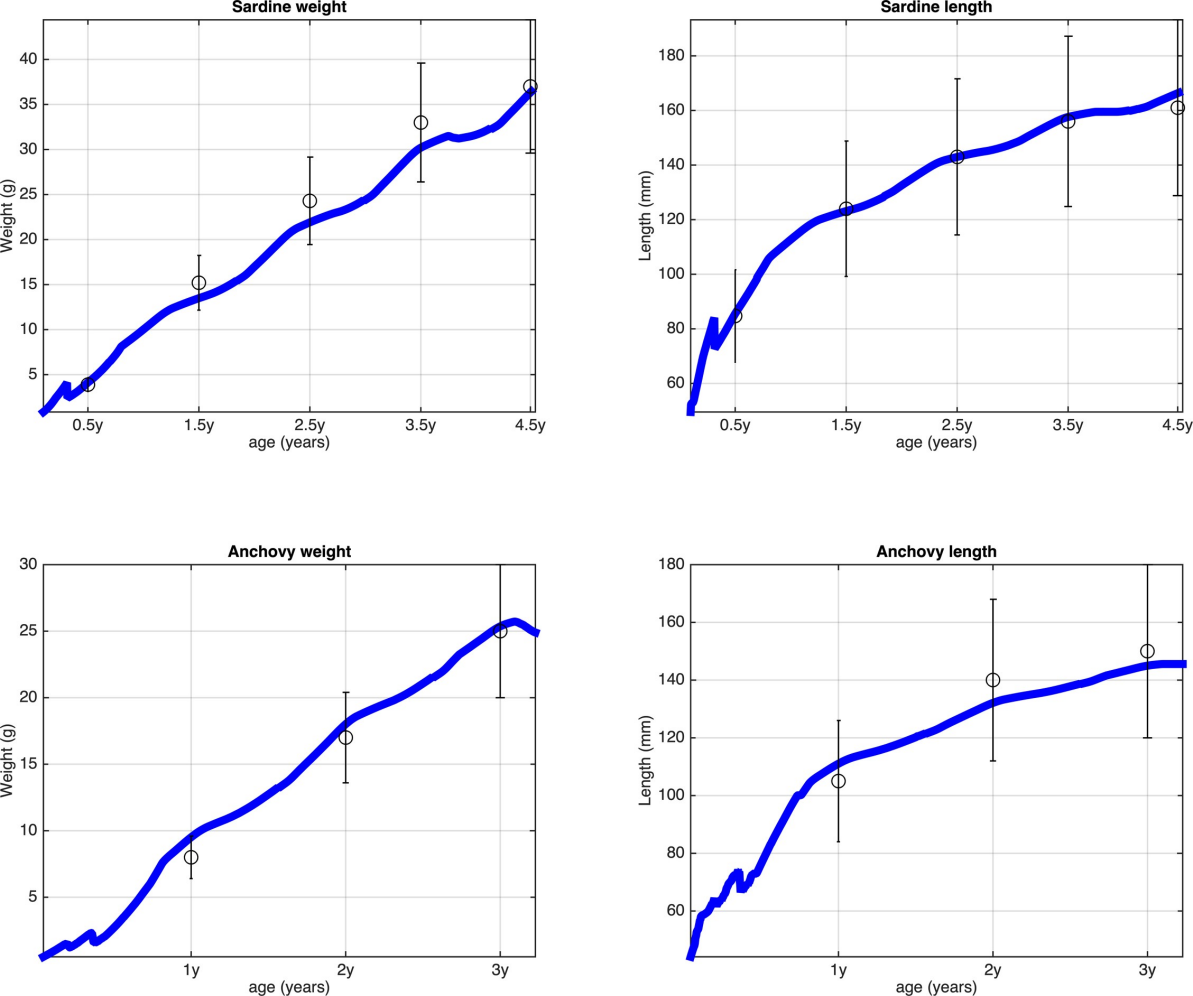
Evolution of mean weight and mean length of fish, calibrated using the genetic algorithm and mean weight- and length-at-age of adult fish (±SD) estimated from samples collected during the acoustic and egg production surveys in the North Aegean Sea, 2003–2008 [42].

The application of the genetic algorithm to tune the half saturation coefficients resulted in growth trajectories that were in close agreement with available lengths- and weights-at-age data from field samples, in both larvae (Fig 6) and adults (Fig 7).

**Fig 8:**
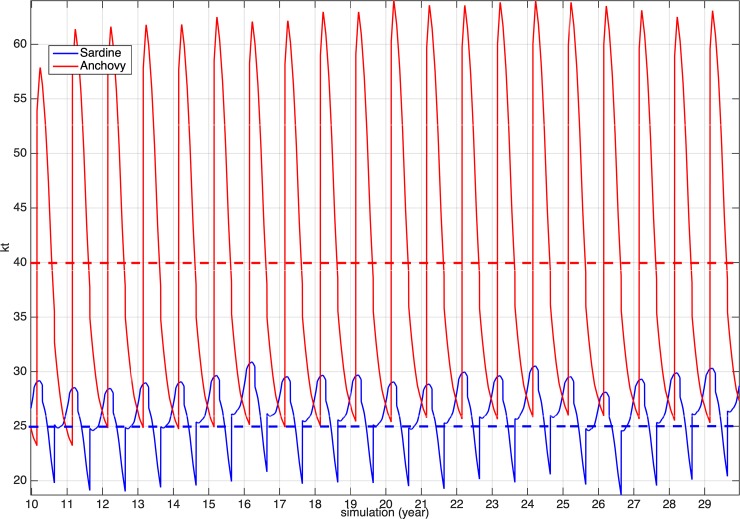
Model-simulated anchovy and sardine biomass. The mean biomasses of the two species in the N. Aegean Sea (based on acoustic data biomass estimations for the period 2003–2008) are also shown.

Finally, after the 10-years spin-up period, the modelled biomasses of the anchovy and sardine populations fluctuated around 40000 t and 25000 t respectively, i.e. the adopted reference biomass values (Fig 8).

**Fig 9:**
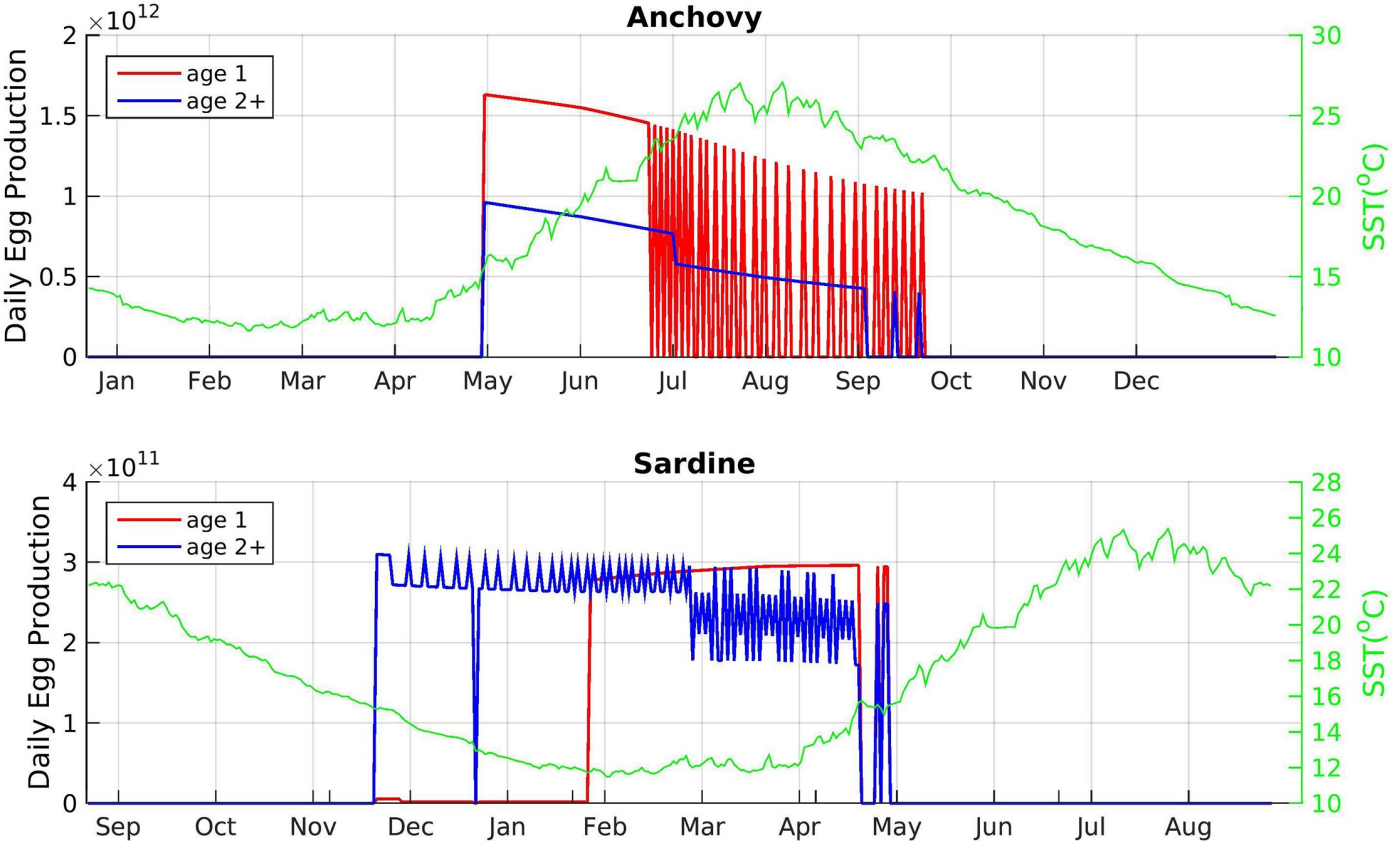
Model-simulated daily egg production (total number of eggs produced by the population) for recruit (age 1) and repeat spawners (age 2+). The seasonal evolution of sea surface temperature (SST) is also shown.

Model outputs regarding the spawning period and daily egg production of the two species were in agreement with known patterns (Fig 9): Anchovy starts spawning in late April and its population continues to release eggs up to late September, but with decreasing numbers, especially after early summer, when SST reaches high values (Fig 4) and the mesozooplankton concentration decreases (Fig 5). No obvious difference in spawning timing/duration was observed between recruit (age-1) and repeat spawners (age 2+) (Fig 9). In sardine, spawning starts in November and lasts until the end of April, i.e. spawning mainly coincides with the period of increase in mesozooplankton concentration (Fig 5). The model also predicts that, in sardine, recruit spawners have a delayed and shorter spawning period than the repeat spawners.

In contrast to expectations [14], the mean values of the ‘capital index’ are much higher in anchovy than in sardine (Table 3). This implies that the sardine in the North Aegean Sea derives most energy for egg production from direct food intake rather than energy stored prior to the spawning period. Indeed, the field estimates of mean monthly condition (Fig 5) indicated that sardine has the lowest somatic weight in autumn prior to the start of its winter spawning season.

**Table 3:** Mean value of the capital index per age class.

	Age 1	Age 2	Age 3	Age 4
Anchovy	0.47	0.71	0.75	-
Sardine	0.006	0.28	0.34	0.09

### Changes in fishery exploitation rates

**Fig 10:**
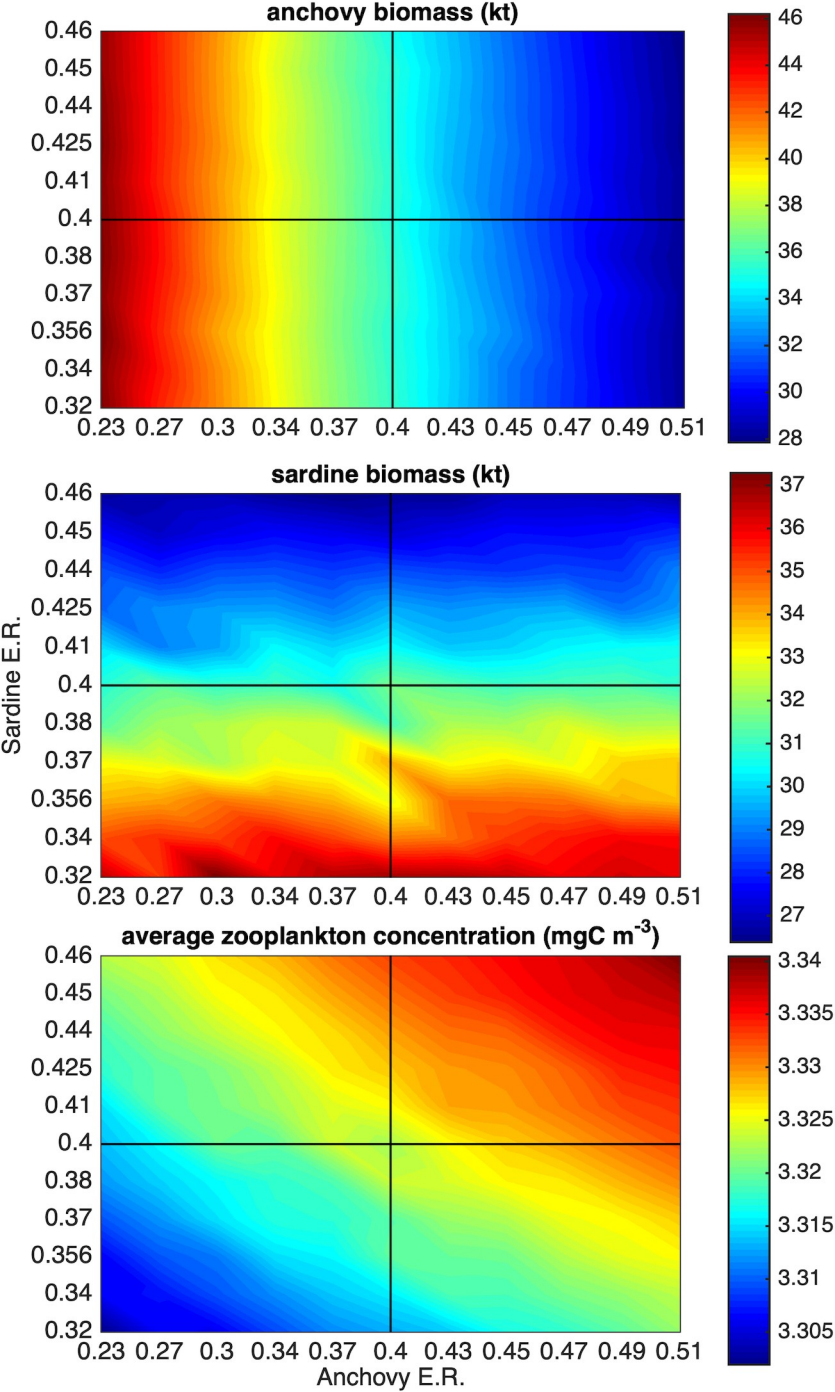
Biomass of anchovy and sardine and mean mesozooplankton concentration for different combinations of exploitation rate (E.R.) of the two species. E.R. = 0.4 is the reference point (maximum sustainable yield proxy) currently used in the management of small pelagic fish stocks in the Mediterranean Sea.

Changing the fishing mortality imposed on the two species, so as to vary the Patterson’s exploitation rate above and below the 0.4 reference point (Fig 10), showed that the biomass of each individual species is relatively insensitive to changes in the exploitation rate of the other species and concomitant changes of its biomass. However, an obvious effect of the combined fishing rates on the two species could be seen on mesozooplankton, which is the fish prey. Sustainable exploitation of both species (E.R. <0.4) results in the decrease of mesozooplankton availability and overexploitation (E.R.>0.4) leads to the increase of mesozooplankton concentration.

### Changes in the timing of the fishery closure period

**Fig 11:**
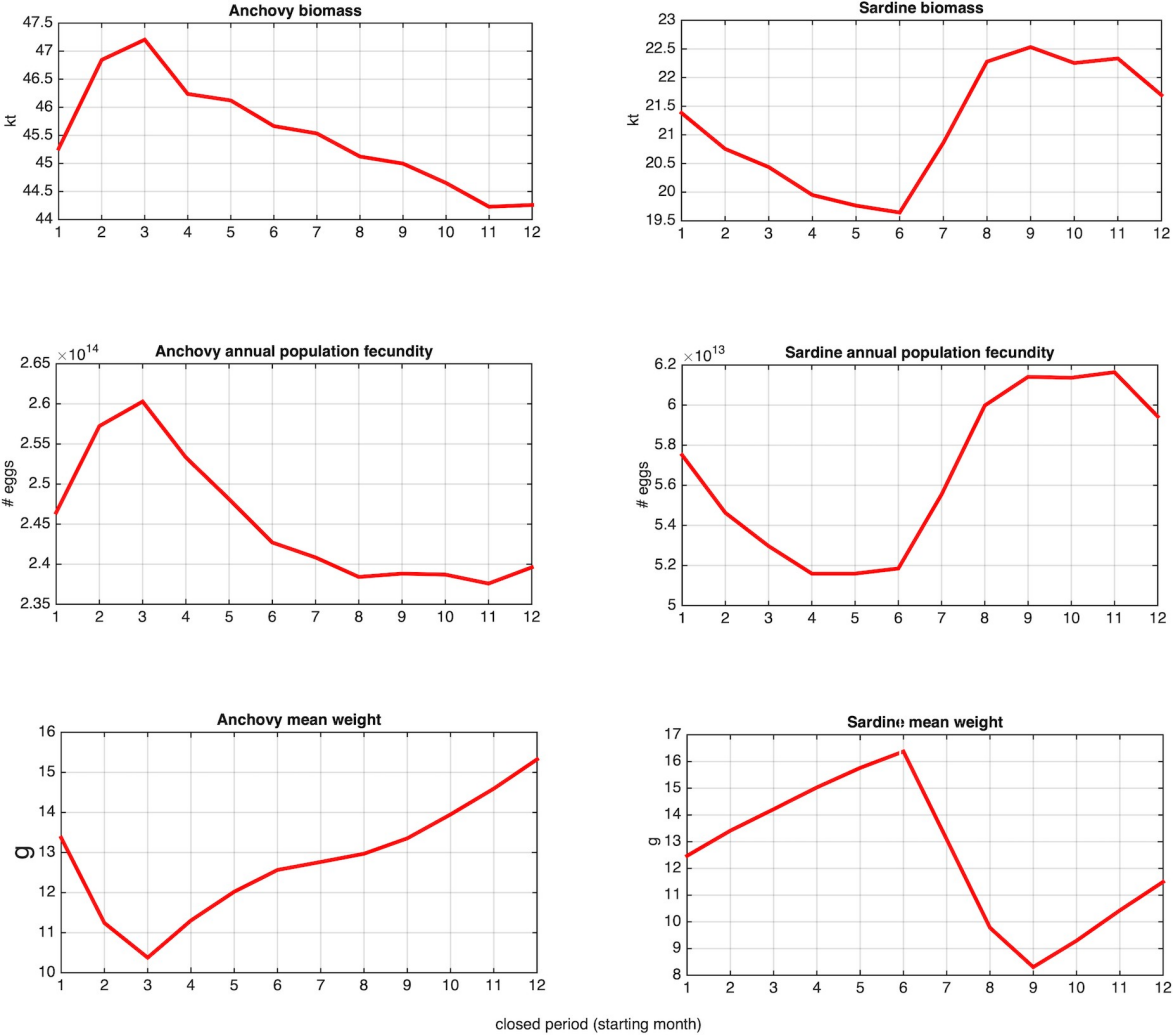
Mean anchovy and sardine biomass (upper panel) and annual population fecundity (middle panel) in relation to the timing of the 2.5 months fishing ban. Months 1, 2, 3,… etc correspond to closed period 15 Jan-Mar, 15 Feb-Apr, 15 Mar-May, …etc. The mean weight of individuals during the respective closed period is also plotted (lower panel).

Shifting the timing of the fishery ban affects the biomass of both species (Fig 11); however suitable timing (i.e., leading to the increase in average biomass) differs between anchovy (spring) and sardine (autumn). In both species, the most favorable closure period is the period of (and around) peak recruitment, as evidenced by the decline of mean fish weight in the population (Fig 11, lower panel). When protecting the recruiting fish prior and/or during the initial phase of their first spawning period population biomass is positively affected, clearly owing to the increased annual population fecundity (Fig 11, middle panel). In other words, due to the numerical dominance of recruit spawners in the population (>70% in both species, not shown), allowing a higher number of them to spawn results in the increase of egg production and the subsequent increase of population biomass.

## Discussion

The full life cycle IBM model developed and evaluated in this paper describes the population dynamics of two species, using a water column model. It can easily be extended to a model that includes more pelagic species (e.g. forage species, predators) and intraguild predation (predation on the eggs of the competing species). Furthermore, with the addition of a movement-migration module, it can become a 3-D fully coupled model, allowing for the direct linking of growth, mortality, movement and spawning processes to the detailed spatial and temporal scales of the hydrodynamic/biogeochemical model (e.g. [13]).

With the exemption of such spatial dimension, our model includes all other processes necessary to simulate growth, egg production and population dynamics and it is two-way coupled with the LTL model. It should be noted here that, in non-upwelling systems like the North Aegean Sea, in which a strong vertical heterogeneity in temperature and zooplankton develops during the thermally stratified period (e.g. Fig 4), it is important to incorporate a diel vertical migration (DVM) behavior in the fish model because temperature and food availability, and consequently consumption and metabolic rates, will change between day and night. In our region anchovy and sardine have a very similar DVM with fish moving above the thermocline during the night and below it, during the day ([33], [34]). The simple vertical migration algorithm developed in [19] and also used here, accounts for the consequences of DVM behavior on consumption and respiration due to thermal stratification and the formation of deep chlorophyll/zooplankton maxima.

In developing the model for sardine we started with the already existing parameterization for anchovy ([18], [19]), changing only those parameters that are known to differ between the two species, i.e. the length-weight relationships, the length ranges of early life stages and number of age classes, but most importantly, their reproductive characteristics, i.e. spawning period, fecundity and egg size. The genetic algorithm applied to tune the bioenergetics model resulted in simulating growth trajectories that were very close to size-at-age data from the field.

Genetic algorithms have previously been applied by [66] and [67] for tuning the weights of an artificial neural network used for habitat choice, energy allocation and spawning strategy/spawning migration, respectively. In our study, tuning the bioenergetics model involved the adjustment of the half saturation parameters so that the simulated fish growth matched the mean size-at-age data estimated from field samples. This computationally demanding process was effectively tackled by a heuristic optimization technique based on a genetic algorithm. The deployed algorithm minimizes the execution time and produces solutions close to optimal (i.e. if not the overall best from all feasible solutions, it finds one very close to the best). Our experimentation showed that the best tuning is achieved, when applying the process sequentially from the younger to older stage/age rather than when concurrently considering all stages/ages, which can be attributed to the dependence of each life stage on previous growth history. The deployed method was very effective and accurate and depending on available hardware, it could be applied to tune more fish processes such as population parameters and temperature dependence.

When calibrating parameters such as the half saturation constants one assumes that food consumption is adapted to local prey availability [26]. Given the similarity of the two species in the North Aegean Sea, e.g. the similar lengths-/weights-at-age (Fig 7), as well as the lack of information on how temperature affects their energetic rates, we adopted the same parameterization for temperature dependences, except for the optimum temperatures for food consumption, which were stage specific and were assumed to be close to the average temperature of the larval, juvenile and adult habitats [6]. In this logic, the major difference between the two species was that the optimum temperature for consumption was lower in sardine larvae (that grow in winter-spring) and higher for anchovy larvae (that grow in summer). This is somewhat consistent with the ‘optimal growth temperature hypothesis’: [68] demonstrated that the larvae of anchovy and sardine have different temperature optima for growth in the NW Pacific, which might be an explanation for the anchovy and sardine population alternations in this region.

Field data on somatic condition showed that both anchovy and sardine increase their energy reserves from winter to early summer, when the simulated mesozooplankton concentration is also increasing (Fig 5). However, from mid-summer onwards, somatic condition declines sharply, lagging the modelled mesozooplankton decline by approximately one month. This finding was unexpected. Several other sardine stocks have been shown to increase their condition all along the summer months, exhibiting maximum condition and lipid storage prior to the onset of gonadal maturation in autumn ([28], [14], and references therein). These sardine stocks are mostly capital breeders using primarily stored energy to produce eggs [14]. In contrast, both the observed seasonal variation of somatic condition and the calculation of the capital index from the model simulation suggest that the sardine stock in the North Aegean Sea is closer to the income breeding mode. On the contrary, anchovy, which starts to spawn in a period of increased zooplankton concentration and continues to release eggs in the subsequent period of maximal surface temperatures/sharply decreasing food availability, is primarily a capital breeder. This can be attributed to the peculiar pelagic production cycle and stressful summer temperatures in the oligotrophic Aegean Sea, where the first half of the year (winter-spring) is the period of increasing zooplankton concentration, in contrast to other ecosystems like those inhabited by the Atlantic anchovy and sardine stocks in which the zooplankton concentration is high in spring-summer and very low in the autumn-winter period [26]. Indeed, in the Bay of Biscay, European anchovy is primarily income whereas European sardine, capital breeder [17]. The indications that the North Aegean Sea anchovy is mostly capital breeder contradict an earlier suggestion, based on data from the early 90’s, that it is income breeder [39]. Recent papers suggest that the period of maximal SPF energy storage in the Mediterranean has changed in recent years (from autumn to early summer) probably reflecting a change in the phenology of plankton production ([10], [11]). As shown by the modelling study of [17], and supported by a review paper on fish breeding patterns [38], the capital-income mode can be plastic in many species; fish can move along the capital-income breeding continuum, in response to their physiological condition and the match-mismatch between the production of food and the production of eggs.

The energy allocation and reproduction algorithm developed in this study resulted in spawning periods that were consistent with observed spawning periods of the two species in the Eastern Mediterranean ([37], [14]). In sardine that spawns in the period of increasing zooplankton concentration both the onset and the end of the spawning period is determined by its SST threshold, whereas in anchovy the SST threshold triggers only the onset of the spawning period. The end of spawning simply results from the exhaustion of reserves from the reproductive buffer and energy intake insufficient to meet the needs of maintenance towards the end of summer. It should be noted here that because the model is 1-D, temperature or other thresholds imposed concurrently to all SIs result in the abrupt starting and ending of spawning periods. However, in a 3-D extension of such model, the population egg production is expected to increase and decrease more smoothly due to the spatial heterogeneity in temperature and food (e.g. [19]). The simulated egg production highlighted that sardine age-1 (recruit spawners) start to spawn later than repeat spawners (age 2+) and have a shorter spawning period. This is well documented for sardine in the Eastern Mediterranean [37] and elsewhere ([28] and references therein), but has never been reported for anchovy in the Eastern Mediterranean, nor resulted from the model simulations. This difference can be explained from the contrasted trophic conditions that anchovy and sardine experience before the onset of their first spawning period, i.e. high food concentration in spring vs low in autumn and the subsequent delay in reaching the size at maturity and acquiring energy for reproduction in sardine, but not in anchovy.

The current application assumes that the fraction of energy allocated to reproduction is equal to the fraction allocated to growth. This choice was considered reasonable given the lack of information on energy allocation. Furthermore, there is some evidence that the fraction k is plastic: Tank experiments in Japanese anchovy have demonstrated that energy allocation to reproduction versus growth changes depending on per capita food availability [69].

A 1-D fish model is particularly useful in testing simple management scenarios, especially when spatially explicit fisheries data (e.g. catches, fishing effort) are scant or unreliable, as is the case for Greek and most other Mediterranean stocks [27]. Testing management options with coupled full life cycle models is attractive because the bottom-up control of population fluctuations is directly taken into consideration.

The current model formulation assumed that the diet of the two species is alike. This assumption is supported by recent trophodynamic studies showing that, in contrast to upwelling systems, the daily ration and diet composition of anchovy and sardine in the N. Aegean Sea are remarkably similar ([30], [31], [32]). Although adult sardines ingest phytoplankton as well, the contribution of phytoplankton to dietary carbon is negligible ([70], [31]) and copepods are the main energy source for both species [32]. Despite the high diet overlap and, consequently, food competition between anchovy and sardine in the N. Aegean Sea, the simulations with varying fishing mortalities showed that the biomass of each species was insensitive to changes in the biomass of the other species caused by changes in its exploitation rate. This implies that the simulated mesozooplankton concentration suffices to support the populations of the two species with no obvious trophic competition. Interestingly, what could be seen from the two-species simulations and the two-way coupling of the fish with the lower trophic level model was the top-down control of mesozooplankton by anchovy and sardine. The combined fishing rates on the two species affected the concentration of mesozooplankton, with sustainable exploitations leading to the decrease of mesozooplankton and unsustainable exploitations to its increase. This can eventually have implications for the pelagic ecosystem and fishery in the area. Removal of small pelagic fish may open up ecological space for other species competing with small pelagics for the same zooplankton prey such as jellyfish [71]. For example, in the Benguela system, off the coast of Namibia, overfishing of the sardine stocks in the 60s and 70s led to the outbreak of jellyfish such as *Chrysaora* [72]. Episodes of anchovy *Engraulis encrasicolus* collapse and ctenophore *Mnemiopsis leidyi* explosion occurred in the Black Sea and the Caspian Sea ([73],[74]).

Testing the effect of timing of the 2.5-month closed period highlighted that the most effective timing for both species is the recruitment period which, however, is different for anchovy (spring) and sardine (autumn). The simulations showed that protecting the numerically dominant recruits prior and/or during the initial phase of their first spawning season contributes to the increase in population fecundity and subsequently the increase in population biomass. The current timing of the fishing ban (15 December-February) seems to be more suitable (although not optimal) for sardine and less effective for anchovy. The periods 15 February-April or 15 March-May seems to be the most beneficial for anchovy.

It should be noted here that our simulations were based on fixed natural mortality rates and averaged environmental conditions. However, natural mortalities can vary greatly in time and space in relation to a variety of ecological factors, such as water temperature, fish condition and size of prey and predator stocks. Such variability as well as inter-annual variability in environmental conditions were not considered in this study and the results of the analyses represent average conditions.

Summarizing, the 1D anchovy-sardine IBM developed and calibrated in this study reproduced well the main characteristics of the two stocks in the N. Aegean Sea. The model was useful in assessing the breeding pattern of the stocks as well as the outcomes of simple management measures. The calibration of the anchovy-sardine model to the characteristics of other Mediterranean stocks and the development and application of a 3D version are expected to improve our understanding of the mechanisms controlling variations in abundance, distribution and productivity of SPF populations in the Mediterranean Sea.

## Supporting information

S1 File Estimation of mean monthly somatic condition of anchovy and sardine. (DOCX)
