# Supplementary material for: Development, application and evaluation of a 1-D full life cycle anchovy and sardine model for the North Aegean Sea (Eastern Mediterranean)
Source: PLoS One. 2019 Aug 15;14(8):e0219671. doi: 10.1371/journal.pone.0219671 (PMC6695132; doi:10.1371/journal.pone.0219671)
Supplement: S1 File — (DOCX) [file pone.0219671.s001.docx]

**S1. Supporting Information**

**Estimation of mean monthly somatic condition of anchovy and sardine**

The analysis of somatic condition was based on length (Total length, mm) and weight (Total weight, g) measurements of anchovies (n=11691) and sardines (n=17766) collected on board the commercial purse seine fleet of the Thracian Sea from 2003 to 2008, in the framework of the National Program for fisheries data collection (DCF). To cover all months of the fishing period (March-December) and get an average picture of seasonal changes in somatic condition, data from different years were pooled.

The average monthly somatic condition of anchovies and sardines was estimated using a general linear model approach [42]: Adjusted somatic weights for each month (average somatic condition) were estimated from the ANCOVA model:

log_10_(TW) = a + b_1_×log_10_(TL) + b_2_×(MONTH) + b_3_×(GENDER) + b_4_×(MONTH)×(GENDER) + b_5_×(MONTH)×log_10_(TL) + b_6_×(GENDER)×log_10_(TL) + b_7_×(MONTH)×(GENDER)×log_10_(TL)

where MONTH: the month of sampling (mar, apr, may, ….),

GENDER: male or female, and

a, b_1_, b_2_, …: model coefficients.

Only significant terms (P<0.05) were retained in the final model (backward stepwise selection) and marginal means were estimated at overall average length [42]. For the appropriateness of the general linear model approach in studies of fish condition, see [62] and [75].

The final general linear models for anchovy and sardine are presented in Tables S1 and S2. Estimated marginal means for monthly somatic weight (average somatic condition) are presented in Table S3.

**Table S1.** **Results of the final general linear model for anchovy with total weight (log_10_TW) as dependent variable. MONTH: month of sampling. TL: total length. Estimated marginal means for term MONTH (at overall mean TL) are presented in Table S3.**

|  | SS | df | MS | F | p |
| --- | --- | --- | --- | --- | --- |
| Intercept | 32.783 | 1 | 32.783 | 27550.16 | <0.001 |
| MONTH | 0.171 | 9 | 0.019 | 15.93 | <0.001 |
| MONTH×GENDER | 0.040 | 9 | 0.004 | 3.71 | <0.001 |
| log_10_(TL) | 48.834 | 1 | 48.834 | 41039.35 | <0.001 |
| MONTH×log_10_(TL) | 0.181 | 9 | 0.020 | 16.91 | <0.001 |
| Error | 13.765 | 11568 | 0.001 |  |  |

Adj.-r^2^=0.909

**Table S1.** **Results of the final general linear model for anchovy with total weight (log_10_TW) as dependent variable. MONTH: month of sampling. TL: total length. Estimated marginal means for term MONTH (at overall mean TL) are presented in Table S3.**

|  | SS | df | MS | F | p |
| --- | --- | --- | --- | --- | --- |
| Intercept | 44.358 | 1 | 44.358 | 28194.03 | <0.001 |
| MONTH | 0.920 | 9 | 0.102 | 64.95 | <0.001 |
| GENDER | 0.075 | 1 | 0.075 | 47.35 | <0.001 |
| MONTH×GENDER | 0.069 | 9 | 0.008 | 4.87 | <0.001 |
| log_10_(TL) | 69.511 | 1 | 69.511 | 44180.92 | <0.001 |
| MONTH×log_10_(TL) | 0.898 | 9 | 0.100 | 63.42 | <0.001 |
| GENDER×log_10_(TL) | 0.075 | 1 | 0.075 | 47.44 | <0.001 |
| Error | 27.912 | 17741 | 0.002 |  |  |

Adj.-r^2^=0.890

**Table S3.** **General linear model for anchovy and sardine with total weight (log_10_TW) as dependent variable. Estimated marginal means for term MONTH (at overall mean TL). SE: standard error.**

|  | anchovy | | sardine | |
| --- | --- | --- | --- | --- |
| MONTH | log_10_TW | SE | log_10_TW | SE |
| mar | 1.207 | 0.0013 | 1.318 | 0.0014 |
| apr | 1.237 | 0.0012 | 1.326 | 0.0015 |
| may | 1.235 | 0.0011 | 1.331 | 0.0014 |
| jun | 1.232 | 0.0006 | 1.340 | 0.0007 |
| jul | 1.231 | 0.0015 | 1.365 | 0.0018 |
| aug | 1.218 | 0.0023 | 1.345 | 0.0015 |
| sep | 1.215 | 0.0013 | 1.335 | 0.0012 |
| oct | 1.198 | 0.0010 | 1.315 | 0.0007 |
| nov | 1.198 | 0.0014 | 1.296 | 0.0007 |
| dec | 1.188 | 0.0018 | 1.297 | 0.0008 |

**References**

1. Vila-Gispert, A., and Moreno-Amich, R., 2001. Fish condition analysis by a weighted least squares procedure: testing geographical differences of an endangered Iberian cyprinodontid. Journal of Fish Biology, 58: 1658–1666.
